# Embryotoxic activity of 3C protease of human hepatitis A virus in developing *Danio rerio* embryos

**DOI:** 10.1038/s41598-021-97641-5

**Published:** 2021-09-14

**Authors:** Polina I. Selina, Maria A. Karaseva, Alexey A. Komissarov, Dina R. Safina, Nataliya A. Lunina, Marina P. Roschina, Eugene D. Sverdlov, Ilya V. Demidyuk, Sergey V. Kostrov

**Affiliations:** Institute of Molecular Genetics of National Research Center, Kurchatov Institute, 123182 Moscow, Russia

**Keywords:** DNA, Enzymes, Proteases

## Abstract

The 3C protease is a key factor in picornavirus-induced pathologies with a comprehensive action on cell targets. However, the effects induced by the enzyme have not been described at the organismic level. Here, the model of developing *Danio rerio* embryos was used to analyze possible toxic effects of the 3C protease of human hepatitis A virus (3Cpro) at the whole-body level. The transient 3Cpro expression had a notable lethal effect and induced a number of specific abnormalities in *Danio rerio* embryos within 24 h. These effects are due to the proteolytic activity of the enzyme. At the same time, the 3Cpro variant with reduced catalytic activity (3Cmut) increased the incidence of embryonic abnormalities; however, this effect was smaller compared to the native enzyme form. While the expression of 3Cmut increased the overall rate of abnormalities, no predominance of specific ones was observed. The data obtained point to a presence significant impact of picornavirus 3Cprotease at the whole-organism level and make contribution to the study of the infectious process caused by human hepatitis A virus.

## Introduction

The pathogen-host interactions are complex and realized at different levels. Analysis of the pathogenic mechanisms makes it possible to expose the pattern common for different agents as well as to reveal promising targets for therapeutic intervention. One should consider that the complex interaction with host cells and tissues can involve pathogen components that affect different targets. The complex of such interactions defines the pathology pattern. In this context, one of the approaches aimed at deciphering the mechanisms of pathology development and searching for promising methods of therapy*,* includes the analysis of possible involvement of individual pathogenic molecules in the interaction with the host. It seems plausible to combine traditional cell models with the organismic level since the cell models cannot properly represent the interaction of pathogenic factors with numerous host cell populations and supracellular systems. Here, we used a model system based on developing *Danio rerio* embryos to analyze possible toxic effects induced by the viral 3C protease at the whole-organism level. This protein is critical in the picornavirus-mediated pathology and seems to have an integral effect on cell targets^1–8^.

Previously, using continuous in vitro cultures it has been demonstrated that the 3C protease can induce different cell death types depending on picornavirus species^9–18^; however, its impact has not been studied at the whole-organism level. We analyzed the embryotoxic effect of picornavirus 3C protease using the enzyme of human hepatitis A virus (3Cpro) inducing caspase-independent cell death in vitro^11^.

## Results

### Expression constructs

**Figure 1:**
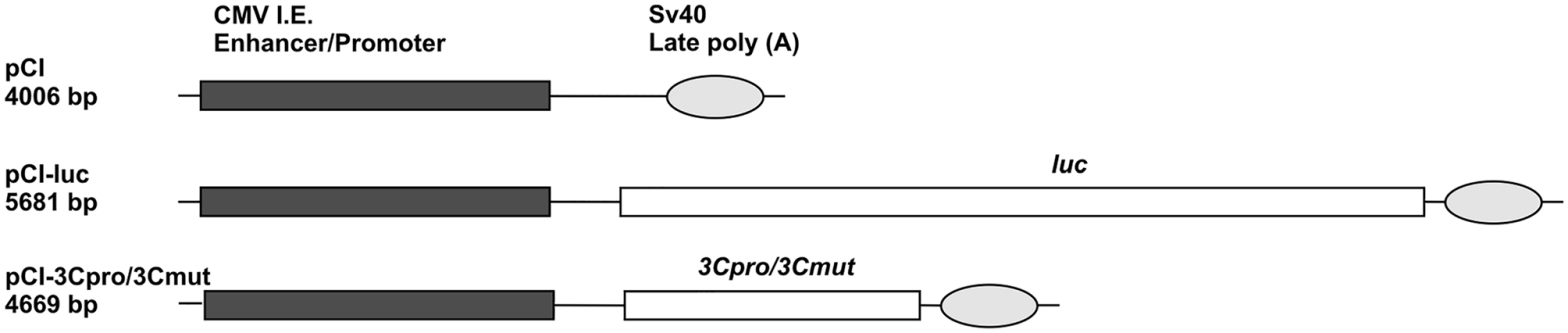
Structure of expression cassettes in genetic constructs used. CMV Enhancer/Promoter, enhancer and promoter of immediate early genes of human cytomegalovirus; *luc*, reporter luciferase gene of firefly *Photinus pyrales*; *3Cpro* and *3Cmut*, genes of native and inactivated mutant 3C protease of human hepatitis A virus, respectively. SV40late poly(A), polyadenylation signal of SV40 late genes.

The possible embryotoxic activity of 3Cpro was evaluated by inoculation of the experimental and control constructs into the *Danio rerio* yolk of fertilized eggs prior to the fist division of cleavage period (within 30 min after fertilization) at 0.3 to 33 attomoles, after which the embryonic mortality and developmental abnormalities were evaluated. A set of plasmids including pCI and derived vectors pCI-3Cpro, pCI-3Cmut, and pCI-luc, the expression cassette of which contained the native 3Cpro gene, mutant 3Cmut one, or *Photinus pyralis* luciferase gene was used (Fig. 1). The mutant 3C protease had the Cys172/Ala substitution in the active site^11,19^.

### Validation of expression activity of genetic constructs

**Figure 2:**
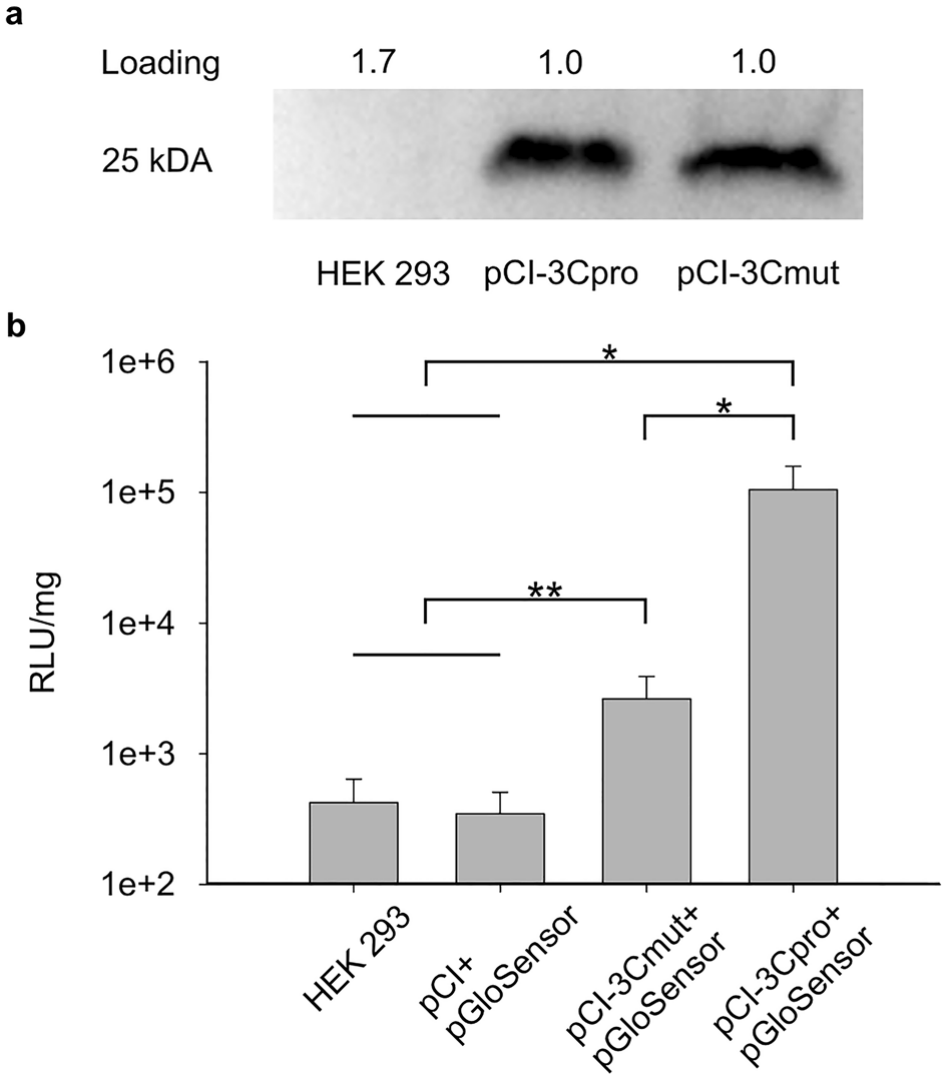
Accumulation of target proteins in HEK293 cells. (**a**) Western blot analysis of 3Cpro in HEK293 cells 24 h after transfection with pCI-3Cpro or pCI-3Cmut. Each lane contained a lysate of 200 000 cells. “Loading” indicates the relative total protein in the lane evaluated by the Stain-Free technique. Full-length blot image is presented in Supplementary Fig. S1. (**b**) Luciferase activity in lysates of HEK293 cells 15 h after transfection with mixture of pCI, pCI-3Cpro, or pCI-3Cmut with pGloSensor-30F-LRTQS (pGloSensor). Ordinate, relative luminescence units (RLU) normalized to 1 mg of total protein in cell lysates. The values were averaged for two independent experiments, in which three replicates were performed. Error bars indicate standard deviation (SD), *p < 0.001; **p < 0.05.

The expression efficiency of pCI-luc was described previously^20^. The capacity of the generated vectors to synthesize the target proteins was evaluated by transfecting HEK293 cells with pCI-3Cpro and pCI-3Cmut. The data obtained indicate equal native and mutant 3Cpro accumulation in cells transfected with the constructed plasmids (Fig. 2a). Thus, pCI-3Cpro and pCI-3Cmut provide for the synthesis of target proteins.

The proteolytic activity of 3Cpro was detected using the GloSensor Technology, which is based on the formation of active luciferase after proteolytic hydrolysis of the intramolecular spacer (Fig. 2b). The used vector pGloSensor-30F-LRTQS encodes a biosensor with the LRTQS sequence in the spacer sensitive to cleavage by 3C protease of the human hepatitis A virus^19,21^. Our analysis demonstrated increased luminescence in lysate of cells cotransfected with pCI-3Cpro and pGloSensor-30F-LRTQS. The data obtained indicate the synthesis of functionally active 3C protease in HEK293 cells directed by pCI-3Cpro. At the same time, cells cotransfected with pCI-3Cmut и pGloSensor-30F-LRTQS also demonstrated elevated luciferase activity relative to control, which amounted to about 2% of that after cotransfection with pCI-3Cpro and pGloSensor-30F-LRTQS. Thus, the data obtained from the assay can indicate the residual proteolytic activity of the mutant enzyme.

### Evaluation of the effect of the vectors introduced into *Danio rerio* embryos on their development

The impact of the analyzed vectors on embryonic development was considered within 48 h after the injection. It should be noted that the mortality of control (uninjected) embryos within the first 24 h reaches 20% on average and remains largely unaltered later. At the same time, the injection of the buffer solution does not significantly decrease the embryo survival rate relative to uninjected control. Thus, the injection per se has no notable effect on the embryo survival rate under experimental conditions.

**Figure 3:**
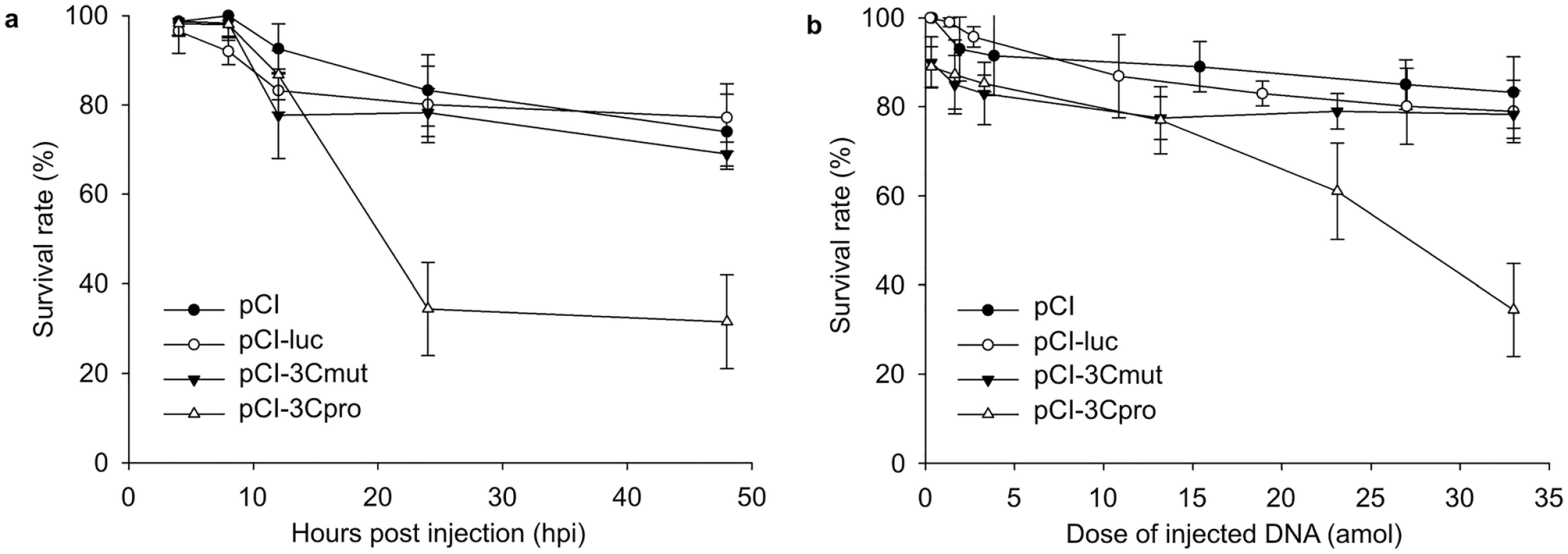
Survival of *Danio rerio* after the injection of DNA constructs. (**a**) Abscissa, time after injection, h; ordinate, number of survived embryos in animal groups injected with 33 attomoles of pCI, pCI-luc, pCI-3Cmut, and pCI-3Cpro. The rate of survived embryos was calculated relative to the number of injected fertilized eggs in each experiment minus the proportion of dead control embryos injected with the buffer solution. Significant (*p* < 0.001) effects induced by pCI-3Cpro relative to pCI, pCI-luc, and pCI-3Cmut were observed 24 and 48 h after injection. The survival rates for each vector used are given as the mean for six independent experiments each with 50 injected fertilized eggs ± SD. (**b**) Abscissa, DNA quantity (attomoles) of injected pCI, pCI-luc, pCI-3Cmut, and pCI-3Cpro. Ordinate, the survival rate of embryos 24 h after DNA injection. The rate of survived embryos was calculated relative to the number of injected fertilized eggs in each experiment minus the proportion of dead control embryos injected with the buffer solution. Significant differences were observed after the injection of 23 (*p* < 0.05) and 33 attomoles (*p* < 0.001) of pCI-3Cpro relative to pCI, pCI-luc, and pCI-3Cmut. The survival rates for each dose of administered vector DNA are given as the mean for six independent experiments each with 50 injected fertilized eggs ± SD.

The data obtained (Fig. 3a) indicate a similar effect of 33 attomoles of pCI, pCI-luc, or pCI-3Cmut introduction, decreasing the embryo survival rate by ~ 25% 48 h after injection*.* At the same time, the injection of pCI-3Cpro had a more pronounced effect and decreased the survival rate by 70%.

The analysis of embryo survival as a function of DNA doses (Fig. 3b) also indicates that the injection of pCI-3Cmut as well as control plasmids pCI and pCI-luc has a similar effect on the survival rate. The injection of 2–4 attomoles of these vectors increased the embryonic mortality by 10–15% relative to control injected by the buffer solution, and this rate did not significantly increase with the DNA quantity raising within the studied dose range. Obtained data indicate that in these cases, the observed embryotoxic effect does not depend on the nature of the expressed gene and, apparently, can be associated with the response of the embryo's body to the introduction of exogenous DNA molecules.

At the same time, the injection of pCI-3Cpro induces a more pronounced embryotoxic effect. The pCI-3Cpro dose of 13 attomoles induced no substantial decrease in the embryo survival rate relative to pCI-3Cmut. However, 33 attomoles of pCI-3Cpro per embryo increased the mortality by 45% relative to control (pCI-3Cmut and pCI), which points to the toxic effect of 3Cpro at the whole-organism level relative to its enzymatic activity. Apparently, this effect requires the vector entry into a fairly large number of embryonic cells and/or the accumulation of significant quantities of catalytically active 3Cpro.

Further data on the specific embryotoxic effect of the 3C protease were obtained by analyzing the rates of developmental abnormalities in *Danio rerio* embryos. Abnormalities in the early *Danio rerio* development are not uncommon. In the AB line used, developmental abnormalities were observed 4 h after fertilization in about 20% of uninjected control embryos. Most of them (~ 70%) are non-developing fertilized eggs dying within 20 h. The rest of the embryos continue to develop but demonstrate lack, deformed or swollen body parts, spinal curvatures, and other abnormalities 24 h after fertilization. At the same time, about 10% of embryos showing normal development 4 h after fertilization had similar abnormalities by 24 h. Thus, the abnormal body structure was observed on average in 14% of animals 24 h after fertilization. The injection of the buffer solution increased the incidence of abnormalities by ~ 10%.

**Figure 4:**
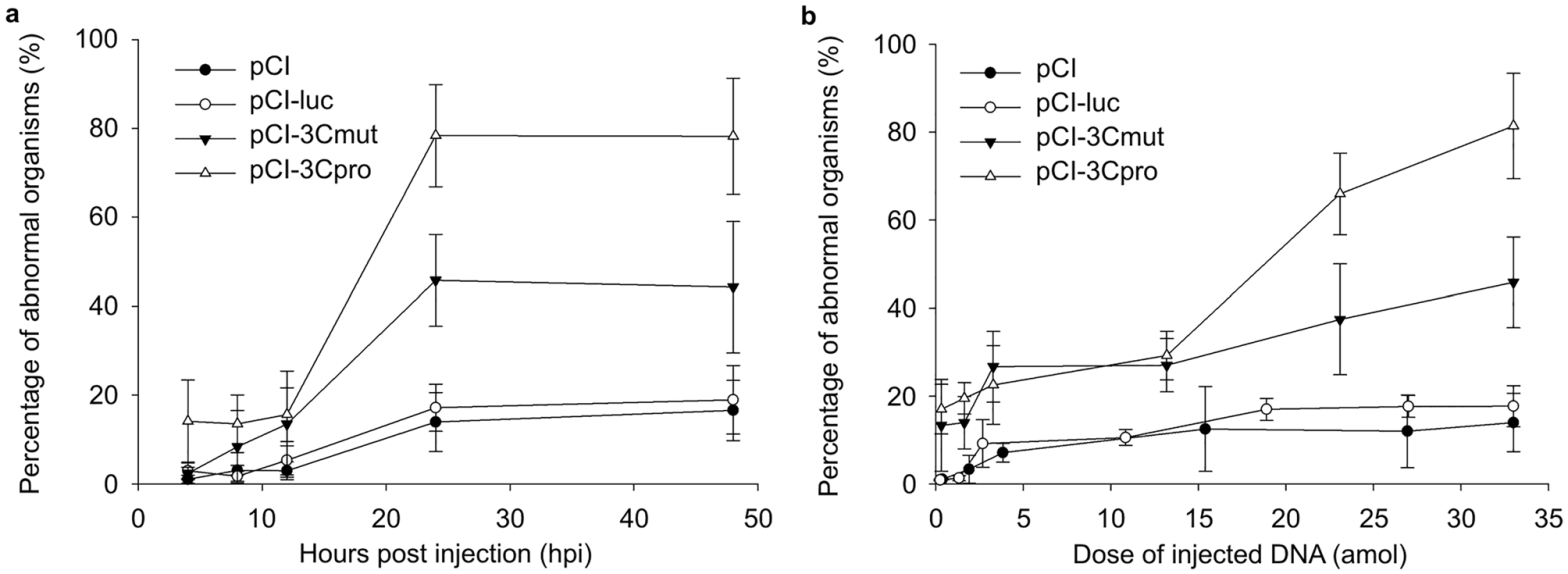
Developmental abnormalities in *Danio rerio* embryos injected with DNA vectors. (**a**) Abscissa, time (h) after injection of 33 attomoles of pCI, pCI-luc, pCI-3Cmut, and pCI-3Cpro. Ordinate, the number of abnormal embryos relative to those survived by the time point minus the proportion of abnormal control embryos injected with the buffer solution. Significant differences in the number of abnormal embryos after the injection of pCI-3Cpro and pCI-3Cmut relative to control vectors pCI и pCI-luc were observed 24 (*p* < 0.001) and 48 (*p* < 0.01) h after injection. The significance of differences in the number of abnormal embryos in the groups injected with pCI-3Cpro and pCI-3Cmut after 24 and 48 h was *p* < 0.01. The percentage of abnormal organisms for each vector used are given as the mean for six independent experiments each with 50 injected fertilized eggs ± SD. (**b**) Abscissa, the dose of injected DNA (attomoles) of pCI, pCI-luc, pCI-3Cmut, and pCI-3Cpro. Ordinate, the rate of abnormal embryos relative to those survived 24 h after DNA injection minus the proportion of abnormal control embryos injected with the buffer solution. Significant differences in the number of abnormal embryos for pCI-3Cpro and pCI-3Cmut relative to control vectors pCI и pCI-luc was observed after the injection from 3 to 33 attomoles (*p* < 0.05). The significance of differences between the pCI-3Cpro and pCI-3Cmut groups was *p* < 0.01. The percentage of abnormal organisms for each dose of administered vector DNA are given as the mean for six independent experiments each with 50 injected fertilized eggs ± SD.

The injection of the genetic constructs influenced the incidence of embryonic abnormalities (Fig. 4a). At the same time, the impact varied with the plasmid introduced. As against animals injected with the buffer solution, the infusion of 33 attomoles of control pCI and CI-luc increased the proportion of abnormally developing embryos by 15% 24 h after the injection. At the same time, the injection of pCI-3Cmut and pCI-3Cpro induced abnormal development in about 45 and 80% of developing embryos, respectively.

The analysis of the relationship between the incidence of developmental abnormalities and the DNA dose introduced (Fig. 4b) shows largely similar patterns for pCI and pCI-luc. DNA injection at 0.3–2 attomoles per embryo induced no increase in the abnormality rate relative to control injected with the buffer solution. However, the injection of 3–4 attomoles per embryo increased the incidence of abnormalities, which peaked 24 h after injection (~ 10% higher than control).

Notice that a further increase in the plasmid dose from 3 to 33 attomoles did not notably increase the incidence of observed abnormalities. Thus, the threshold dose for pCI and pCI-luc inducing no more increase in the rate of abnormalities (3–4 attomoles) corresponds to that for their impact on the embryo survival rate (Fig. 3a).

In the case of pCI-3Cpro and pCI-3Cmut, a significant increase in the number of abnormal embryos (~ 15%) relative to animals injected with pCI and pCI-luc was observed at the DNA dose of 2–4 attomoles; no difference was observed between pCI-3Cpro and pCI-3Cmut. As the dose increased to 33 attomoles, pCI-3Cpro induced a higher number of abnormalities than pCI-3Cmut. The difference in the number of abnormal embryos was about 35% 24 h after the injection (Fig. 4a; Fig. 4b).

Thus, an embryotoxic effect much higher than the control level is observed as both native 3Cpro and its mutant variant accumulate in the embryo; however, the toxic effect of the protein is much higher in the former case.

**Figure 5:**
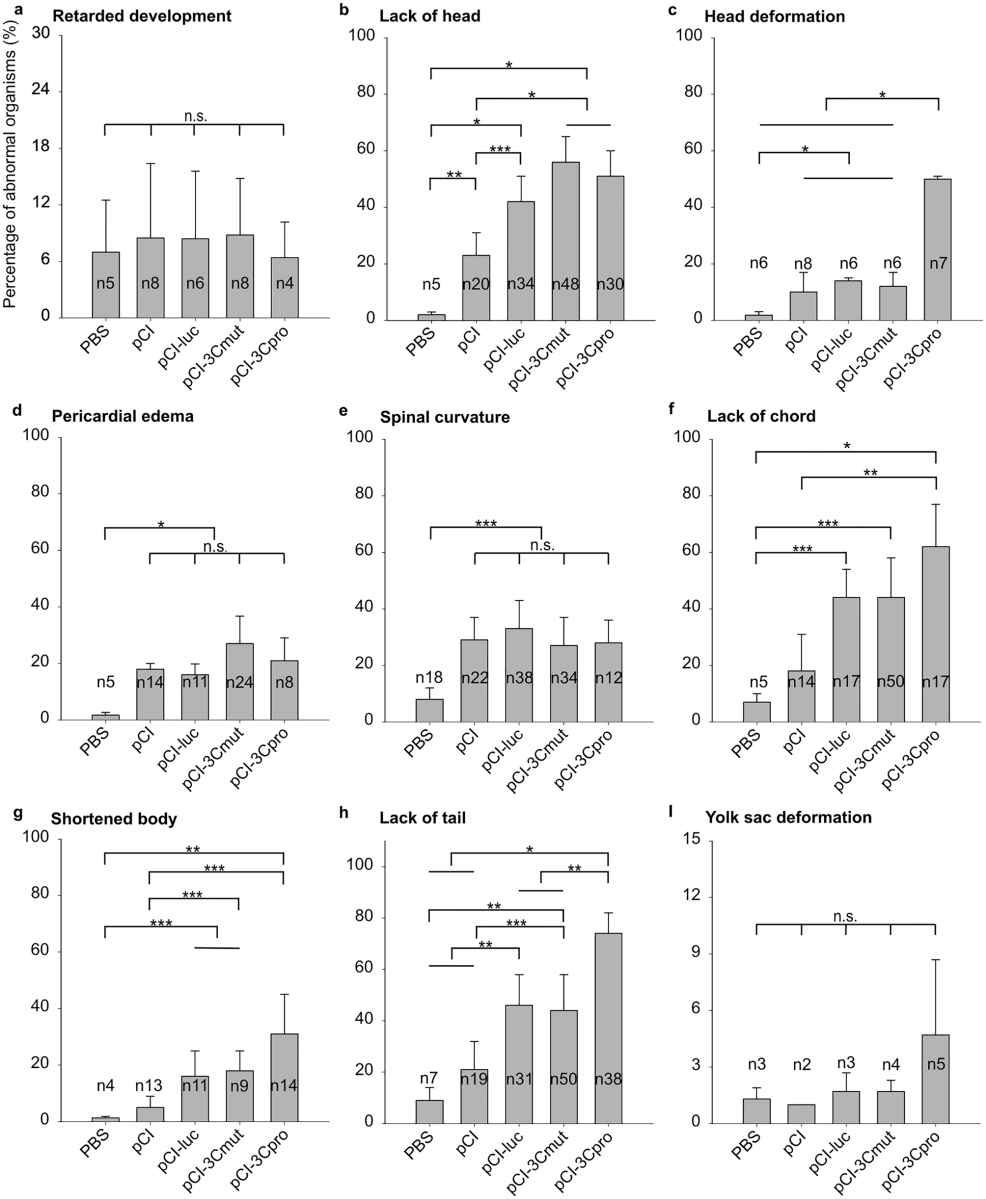
Main abnormalities revealed in developing *Danio rerio* embryos. Data are given for embryo injected with PBS or 33 attomoles of pCI, pCI-luc, pCI-3Cmut, and pCI-3Cpro. The incidence of each developmental abnormality (ordinate) was calculated relative to the total number of abnormal embryos observed 24 h after injection minus the proportion of control (uninjected) embryos with the same abnormality. Values are represented as mean of six independent experiments. Error bars indicate standard deviation (SD). The number of embryos analyzed (n) is specified in the figure, *p < 0.001; **p < 0.01; ***p < 0.05; *n.s.* not significant for all columns in the specified range.

The induction of specific or nonspecific early developmental abnormalities by 3Cpro expression can be evaluated by comparing the rates of particular embryonic abnormalities in the experimental and control groups (Fig. 5). The most common abnormalities in the used AB line include development delay; abnormal development of the head, heart, and tail; chord curvature or achordia; and shortened body. The same embryo can have several abnormalities at the same time^22,23^.

It should be noted that substantial data variation typical of whole-organism systems complicates the positive identification of weak effects. Still, the data obtained reveal certain patterns. The injection per se likely increases the proportion of embryos with retarded development (~ 10%) (Fig. 5a). At the same time, the injection of exogenous DNA increases the incidence of a wide range of abnormalities. The injection of all analyzed genetic constructs significantly increased the number of acephalic embryos as well as those with head developmental abnormalities (Fig. 5b,c), pericardial edema (Fig. 5d), and spinal curvature (Fig. 5e). The buffer injection induced no comparable effect. At the same time, the injection of pCI-luc, pCI-3Cpro, or pCI-3Cmut expressing alien proteins further increased the incidence of abnormalities such as embryos without the head or notochord (Fig. 5f) or shortened body (Fig. 5g). This effect can be attributed to the emergence of heterologous proteins in embryonic cells sensitive to the protein composition.

Several patterns of embryonic disorders associated with the expression of native 3C protease can be identified. The most striking is the deformed head (Fig. 5c). The number of embryos with this abnormality substantially increases after the injection of pCI-3Cpro, while other plasmids including pCI-3Cmut providing for the expression of the mutant enzyme have no such effect. Moreover, the expression of the native protease increased the incidence of achordate (Fig. 5f) and tailless embryos (Fig. 5h). Thus, the 3C protease of the human hepatitis A virus can specifically induce certain abnormalities in embryonic development, and this effect is directly linked to the proteolytic activity of the enzyme.

## Discussion

The model of developing *Danio rerio* embryos allowed us to discover the embryotoxic effect of the 3C protease of the human hepatitis A virus. The introduction of the genetic construct providing for the synthesis of 3Cpro into fertilized eggs killed most embryos within 24 h. The injection of a similar construct expressing an enzyme variant with reduced proteolytic activity had no such effect; hence, the lethal effect of the 3Cpro protease at the whole-body level is due to its proteolytic activity.

The accumulation of 3Cpro in the survived embryos increased the rate of their developmental abnormalities. The expression of the mutant protease increased the rate of abnormalities with no preference for particular ones. At the same time, the effect of the 3Cpro proteolytic activity on the induction of certain embryonic abnormalities has been revealed. These primarily included irregular-shaped head and notochord or tail loss in the embryos. Thus, 3C protease of the human hepatitis A virus can induce a number of specific developmental abnormalities.

In this context, it may be assumed that the modified enzymatic properties of 3Cpro induced by single mutation Cys172/Ala can influence the range of cellular targets of the protease, which is reflected in the pattern of the embryotoxic effect at the body level and underlies the difference in the effects induced by pCI-3Cpro and pCI-3Cmut.

The molecular mechanisms underlying the embryotoxic effect of 3Cpro are currently unclear. Picornavirus 3C protease can cleave various cellular proteins including regulatory factors, which can affect a wide range of biochemical processes^1–8^. It is worth mentioning that 3C protease activity induces cell death^9–18^, and thus can be considered as the key factor of viral cytotoxicity.

Previously, we demonstrated that the catalytic activity of 3C protease of the human hepatitis A virus can induce cell death through a caspase-independent mechanism^11^. It is possible that the 3Cpro cytotoxicity can underlie the observed embryotoxic effects. The genetic constructs expressing 3Cpro are injected at early embryonic stages, which can provide for the death of cell groups required for further correct body development. It is possible that the pattern of observed abnormalities in survived animals can result from different capacity of embryonic cells to absorb vector DNA from the interstitial space or to their different susceptibility to the 3Cpro-induced cell death.

The data obtained indicate that the injection of plasmid DNA can have a direct embryotoxic effect on the embryo survival rate and their developmental abnormalities. The injection of 2–4 attomoles of pCI, pCI-luc, or pCI-3Cmut increased embryonic mortality by 10–15% relative to control injected with the buffer solution, and this rate remained unaltered as the quantity of injected DNA increased to 33 attomoles. Thus, 2–4 attomoles is likely the threshold dose inducing the lethal response of the embryo to exogenous DNA.

Similar dose dependence is observed for the rate of developmental abnormalities. The injection of about 3–4 attomoles of pCI or pCI-luc per embryo increased the incidence of abnormalities, which peaked 24 h after the injection and was 10–15% higher than that after buffer injection. Further increase in the dose of the plasmids up to 33 attomoles did not significantly increase the incidence of the observed abnormalities. Thus, the threshold DNA doses increasing embryonic mortality as well as the incidence of developmental abnormalities are virtually the same.

It is possible that both processes have a common underground, and it is the appearance of abnormalities in the development of embryos that leads either to the rapid death of animals, or, in more "light" versions, is found in the experiment in the form of disorders in the development of certain organs. These effects are likely due to the functioning of the embryo systems recognizing exogenous DNA as a pathogenic signal.

In this case, our experiments can detect a system providing for programmed body death. In terms of population conservation, the conservation of a particular organism is not always critical or even significant. Under certain conditions, the elimination of individuals can be beneficial for the population. A clear example of such conditions is an infection of a highly dense population, in particular, abundant embryos in egg clutches of fish, mollusks, amphibians, insects, etc.

The elimination of infected individuals in the early infection stages is clearly beneficial for the population. However, this strategy assumes the existence of mechanisms for programmed body death. The model system used in this study essentially simulates an infection attack of a population during early development. The data obtained point to a mechanism of body suicide triggered by exogenous DNA in the extracellular space of the embryo. At the same time, such a program is realized apparently by developmental abnormalities induced by exogenous DNA concentrations exceeding the threshold.

In summary, the model of developing *Danio rerio* embryos was used to demonstrate for the first time the embryotoxic effect of 3C protease of the human hepatitis A virus at the whole-body level related to the induction of animal death and developmental abnormalities. The data obtained point to a presence significant impact of picornavirus 3Cprotease at the whole-organism level and make contribution to the study of the infectious process caused by human hepatitis A virus. In addition, quantitative data describe the direct embryotoxic effect of exogenous DNA, which can indicate a system providing for the programmed organism death as DNA of infection agents appear in the extracellular space of the embryo.

## Materials and methods

### Vector constructs

The expression vector pCI (Promega, United States) and the derived genetic constructs pCI-3Cpro, pCI-3Cmut, pCI-luc were used; the latter was constructed previously^20^. The proteolytic activity of 3Cpro was analyzed using pGloSensor-30F-LRTQS.

### Construction of pCI-3Cpro and pCI-3Cmut

The 3C protease gene of human hepatitis A virus strain HAS-15 was amplified from pBI-EGFP/3C^11^ using primers with EcoRI (3C-dir: 5’-GACTGAATTCGCCACCATGTCAACTCTAGAAATAGCAGG) and KpnI (3C-rev: 5’-CAACGGTACCTTACTGACTTTCAATTTTCTTATCAATG) sites. The primers were synthesized by Evrogen (Russia). The reaction mixture (25 µl) contained 100 ng of DNA template, 0.3 mM deoxynucleotide triphosphate each (Sileks, Russia), 1 U Pfu DNA polymerase, and the corresponding buffer (20 mM Tris–HCl, 10 mM KCl, 10 mM (NH_4_)_2_SO_4_, 2 mM MgSO_4_, and 0.1% Triton X-100, pH 8.8). Polymerase chain reaction was conducted in a MiniCycler (BioRad, United States) using the following program: 94 °C, 3 min; 25 cycles of 94 °C, 30 s; 59 °C, 1 min; 72 °C, 1 min 24 s; and 72 °C, 15 min. The amplification products were fractionated by horizontal electrophoresis in 1% agarose gel and the target fragment was isolated using the Cleanup Mini kit (Evrogen, Russia). The obtained fragment was digested with EcoRI and KpnI and cloned into the same sites of pCI to yield pCI-3Cpro. A similar approach was used to construct pCI-3Cmut using pBI-EGFP/3Cmut^11^ as the template. The enzymes and buffer solutions were purchased from SibEnzyme (Russia). The structure of the constructed vectors was confirmed by sequencing.

### Construction of pGloSensor-30F-LRTQS

Vector pGloSensor-30F-LRTQS including the hydrolytic site LRTQS sensitive to 3C protease of the human hepatitis A virus^19,21^ was generated from pGloSensor-30F-DEVDG (Promega, United States) using the GATCCCTGAGAACCCAGTCA and AGCTTGACTGGGTTCTCAGG oligonucleotides (Evrogen, Russia). The oligonucleotides mixture (10 µM each) was heated to 95 °C and slowly cooled to room temperature. The resulting DNA duplex was cloned into the BamHI and HindIII sites of pGloSensor-30F-DEVDG.

### HEK293 cell culture and transfection

Human embryonic kidney cells HEK293 (obtained from the Russian Collection of Cell Cultures, St. Petersburg, Russia) was cultured in DMEM/F12 (Paneco, Russia) supplemented with 10% fetal bovine serum (GE Healthcare, United States) and 0.3 mg/ml glutamine (MP Biomedicals, United States) at 37 °C under 5% CO_2_.

Cells were transfected with pCI-3Cpro or pCI-3Cmut using the transfection reagent TurboFect (Thermo Fisher Scientific, United States) for analysis of protein accumulation in transfected cells and were co-transfected with mixture of pCI, pCI-3Cpro, or pCI-3Cmut with pGloSensor-30F-LRTQS using the transfection reagent Lipofectamine 2000 (Thermo Fisher Scientific, United States) according to the manufacturer’s protocol. Mass fraction of vector pGloSensor-30F-LRTQS was 50% in the transfection mixture.

### Luciferase activity assay

HEK293 cells were incubated for 15 h after transfection. Cells were lysed and luciferase activity was assayed in the lysates following the recommendations for the Luciferase Assay System (Promega, United States) using a 96-well solid white polystyrene microplates (Corning, United States) and an Infinite M200 PRO microplate reader (Tecan, Switzerland). The substrate solution contained 1 mM D-luciferin from *Photinus pyralis* (Promega, United States), 25 mM Tris–phosphate, 50 mM 2-mercaptoethanol, 2.5 mM ethylenediaminetetraacetic acid (EDTA), 10 mM MgSO_4_, (Amresco, United States), and 4 mM deoxyadenosine triphosphate (AppliChem, Germany), pH 7.8.

### Protein quantitation

Total protein was assayed after Bradford with modifications^24,25^ using a 96-well plate (Corning, United States) and a UV/VIS Infinite M200 PRO microplate reader (Tecan, Switzerland). The dye solution contained 0.03% Coomassie G-250 (LOBA Feinchemie, Austria), 5% ethanol, and 10% phosphoric acid (Chimmed, Russia). Bovine IgG (Reanal, Hungary) was used for the calibration curve.

### Analysis of 3C protease accumulation in transfected HEK293 cells

Culture medium from wells with HEK293 cells transfected by pCI, pCI-3Cpro, or pCI-3Cmut plasmids were removed after 24 h of transfection and cells were incubated in 0.25% trypsin. After 5-min incubation, cells were resuspended in phosphate-buffered saline (PBS) (Biolot, Russia) and centrifuged at 300 g for 5 min. The pellet was resuspended in PBS with 0.02 µl of Protease Inhibitor Cocktail (Sigma-Aldrich, United States). Cells in suspension were counted using a Countess II FL cell counter (Thermo Fisher Scientific, United States). The resulting suspension was diluted with the lysis buffer (125 mM Tris–HCl, pH 6.8, 2% SDS, 5% glycerol, 100 mM 2-mercaptoethanol, and 0.005 M EDTA) to 100,000 cells per 10 µl, and incubated at 95 °C for 5 min.

The lysates were analyzed by polyacrylamide gel electrophoresis (12% TGX Stain-Free FastCast Acrylamide kit, Bio-Rad, United States). Separated proteins were transferred onto a nitrocellulose membrane using a Trans-Blot Turbo apparatus (Bio-Rad, United States). Proteins were visualized on the membrane using the Stain-free system (Bio-Rad, United States) and a ChemiDoc MP imager (Bio-Rad, United States). After overnight blocking in 5% defatted milk (PanReac AppliChem, Spain-Germany) supplemented with 0.1% Tween 20 (Sigma-Aldrich, United States), the membrane was incubated at room temperature for 2 h with 1:300 rabbit serum against 3Cmut protease isolated from the producer strain^26^. After washing three times with 200 ml of PBS containing 0.1 Tween 20, the membrane was incubated with 1:10,000 the secondary goat anti-rabbit antibodies (1 mg/ml) conjugated with horseradish peroxidase (111–035-003, Jackson ImmunoResearch Laboratory, USA) at room temperature for 30 min. Immunopositive bands were visualized by the Clarity Western ECL substrate (Bio-Rad, United States) according to the manufacturer’s recommendations. The chemiluminescent signal was detected using a ChemiDoc MP imager.

### *Danio rerio* maintenance

The wild-type AB strain of *Danio rerio* was used. Fish were kept in a flow-through aquarium (Aqua Schwarz, Germany) at 26–28 °C. The light:dark cycle was 14:10 following the international standard. Fish were fed once a day with nauplii *Artemia salina* (Barrom, Russia) or dry food sera Vipan (Sera, Germany).

The study reported in this paper was conducted in compliance with the ARRIVE guidelines. All experimental procedures were performed in strict accordance with the regulations of the European Convention for the Protection of Vertebrate Animals used for Experimental and other Scientific Purposes (ETS No. 123) and bioethical principles (https://cioms.ch/images/stories/CIOMS/IGP2012.pdf) and were approved by the Research Ethics Committee (#12–2017) of the Institute of Molecular Genetics of the National Research Centre “Kurchatov Institute”.

### DNA microinjection into *Danio rerio* eggs

DNA samples were microinjected into *Danio rerio* embryos at the first cleavage 20 min after fertilization using an M-152 micromanipulator (Narishige, Japan) and an air-pressure injector PicoPump PV820 (World Precision Instruments, United States) under an inverted microscope Olympus IX2-SLP (Olympus, Japan). The samples were injected into the yolk under the formed germinal disc at an angle of 45° to the surface with the embryo to maximize sample delivery into the yolk center.

The capillaries used with the outer diameter of 20 µm were pulled from glass capillaries (BF100-50–10, Sutter Instrument, United States) by a Micropipette puller (Sutter Instrument, United States). A 1 nl sample was injected into an embryo within 2.8 × 100 ms.

Vectors DNA were isolated from transformed *Escherichia coli* TG1 using a Plasmid Miniprep kit (Evrogen, Russia). DNA concentration was determined by spectrophotometry using the extinction coefficient of 0.02 ml/(µg × cm) for double-stranded DNA^27^. The obtained DNA was dissolved in PBS with 0.05% phenol red (Sigma-Aldrich, United Kingdom).

### Quantitation of embryotoxic effect

Six independent experiments with the injection of the buffer solution and each DNA vector dose were conducted, and the mean number of injected embryos was 50, and 50 uninjected embryos were used as a control in each case.

The development of *Danio rerio* embryos was monitored under an inverted microscope Olympus IX2-SLP. Their survival rate as well as the type and number of abnormalities were evaluated for each time point. The proportion of survived embryos was calculated taking the number of injected fertilized eggs as 100% in each experiment. In control, the total number of fertilized eggs was taken as 100%. The number of abnormally developing embryos was calculated relative to the number of animals that survived to the current time point. The proportion of embryos with a particular developmental abnormality was calculated relative to the total number of abnormal embryos at the current time point.

In the case of early developmental stages (4–12 h after injection), the embryo shape, coagulation, and correspondence to the developmental stage were evaluated. Starting from 24 h, the following embryonic abnormalities were considered: cephalic defects (deformed or missing segment), caudal defects (missing segment), circulatory defects (pericardial edema, circulatory and vascular abnormalities), otolith and air-bladder defects, body malformations (spinal curvature, achordia and growth retardation), yolk sac defects (edema or malformations), and retarded development^22,23^.

### Statistical analysis of data obtained

Experimental data were statistically processed by MS Excel 2007 (Microsoft, United States) and SigmaPlot 11.0 (Systat Software, United States). The significance of the difference between groups was evaluated by one-way ANOVA with Tukey’s correction for multiple comparisons*.* Differences were considered significant at *p* < 0.05.

## Supplementary Information

Supplementary Information.
