# Supplementary material for: Embryotoxic activity of 3C protease of human hepatitis A virus in developing Danio rerio embryos
Source: Sci Rep. 2021 Sep 14;11:18196. doi: 10.1038/s41598-021-97641-5 (PMC8440601; doi:10.1038/s41598-021-97641-5)
Supplement: Supplementary file 1 — Supplementary Information. [file 41598_2021_97641_MOESM1_ESM.doc]

**Embryotoxic activity of 3Cprotease of human hepatitis A virus in developing *Danio rerio* embryos**

Polina I. Selina^1*^, Maria A. Karaseva^1^, Alexey A. Komissarov^1^, Dina R. Safina^1^, Nataliya A. Lunina^1^, Marina P. Roschina^1^, Eugene D. Sverdlov^1^, Ilya V. Demidyuk^1^, Sergey V. Kostrov^1^

**Affiliations**

^1^ Institute of Molecular Genetics of National Research Center “Kurchatov Institute”, 123182 Moscow, Russia

**Corresponding author**

^*^Correspondence should be addressed to Polina I. Selina; E-mail: greenapple_35@mail.ru; https://orcid.org/0000-0001-6499-8525

**Supplementary contents**

**
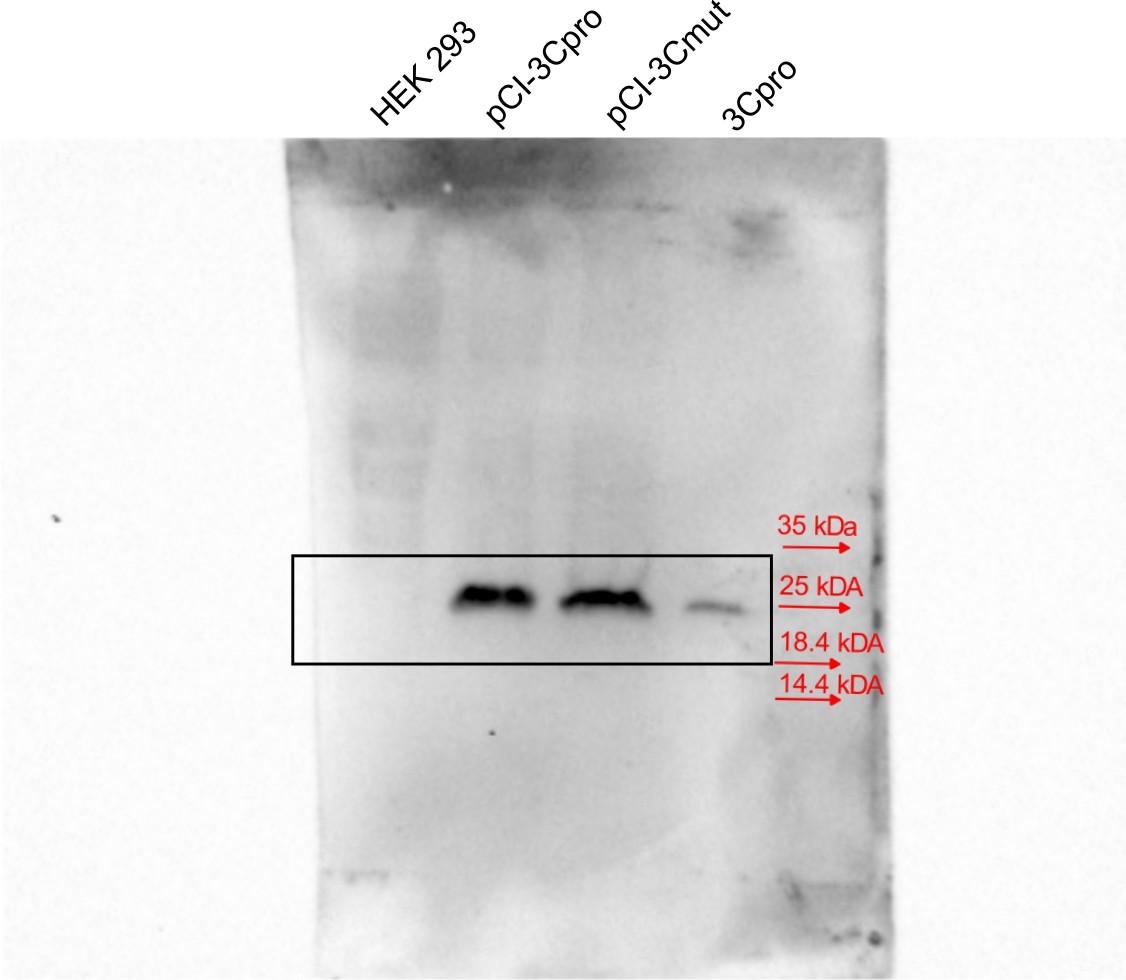
**

**Supplementary figure S1. Full-length blot image from Figure 2a.** The recombinant protease 3Cpro obtained previously [26] was used as a positive control. The 3Cpro lane contained 1 ng of the protein.
